# No Histopathological Evidence of Inflammation Despite Molecular Detection of *Schistosoma* spp. and Sexually Transmitted Pathogens in Placental Parenchyma Specimens with Limited Membrane Sampling from West African Women with Uncomplicated Pregnancies

**DOI:** 10.3390/pathogens14121223

**Published:** 2025-11-30

**Authors:** Jan Theile Suhren, Gunnar Müller, Torsten Feldt, Mathurin Koffi, Samuel Blay Nguah, Carola Bindt, Stephan Ehrhardt, Dana Barthel, Rebecca Hinz, Jana Baum, Lisa Claussen, Harry Tagbor, Stefanie Schoppen, Hagen Frickmann, Kirsten Alexandra Eberhardt

**Affiliations:** 1 Department of Pathology, Bundeswehr Central Hospital Koblenz, 56072 Koblenz, Germany; jantheilesuhren@bundeswehr.org (J.T.S.); gunnarmueller@bundeswehr.org (G.M.); 2 Institute of Pathology, University Clinics of RWTH University, 52074 Aachen, Germany; 3 Department of Gastroenterology, Hepatology and Infectious Diseases, Medical Faculty and University Hospital Düsseldorf, Heinrich Heine University Düsseldorf, 40225 Düsseldorf, Germany; torsten.feldt@med.uni-duesseldorf.de (T.F.);; 4 UFR Environnement-Santé, Laboratoire des Interactions Hôte-Microorganismes-Environnement et Evolution (LIHME), Université Jean Lorougnon GUEDE, Daloa BP 150, Côte d’Ivoire; m9koffi@yahoo.fr; 5 School of Medicine and Dentistry, Kwame Nkrumah University of Science and Technology, Kumasi AK-385-1973, Ghana; sbnguah@gmail.com; 6 Department of Child and Adolescent Psychiatry, Psychotherapy and Psychosomatics, University Medical Center Hamburg, 20251 Hamburg, Germany; bindt@uke.de (C.B.); d.barthel@uke.de (D.B.); 7 Department of Epidemiology, Johns Hopkins Bloomberg School of Public Health, Baltimore, MD 21205, USA; sehrhar6@jhu.edu; 8 Department of Microbiology, Labor Dr. Heidrich & Kollegen, 22081 Hamburg, Germany; hinzr@labor-heidrich.de; 9 Clinical Research Unit, Bernhard Nocht Institute for Tropical Medicine Hamburg, 20359 Hamburg, Germany; jana-baum@hotmail.com; 10 Department of Anaesthesiology and Intensive Care, Asklepios Klinik Altona, 22763 Hamburg, Germany; l.claussen@asklepios.com; 11 School of Medicine, Department of Community Health, University of Health and Allied Sciences, Kumasi, Ho PMB 31, Ghana; htagbor@uhas.edu.gh; 12 Department of Health and Social Science, Hochschule Fresenius, 20148 Hamburg, Germany; stefanie.schoppen@hs-fresenius.de; 13 Department of Microbiology and Hospital Hygiene, Bundeswehr Hospital Hamburg, 22049 Hamburg, Germany; 14 Institute for Medical Microbiology, Virology and Hygiene, University Medicine Rostock, 18057 Rostock, Germany; 15 Department of Tropical Medicine, Bernhard Nocht Institute for Tropical Medicine and I. Department of Medicine, University Medical Center, 20359 Hamburg, Germany

**Keywords:** histopathology, placenta, schistosomiasis, sexually transmitted infection, STI, immunohistochemistry, pregnancy, maternal health

## Abstract

Background: Placental infections caused by *Schistosoma* spp. and sexually transmitted microorganisms can adversely impact pregnancy outcomes. However, the association between molecular detection of these pathogens in placental tissue and corresponding histopathological inflammation remains unclear, particularly in sub-Saharan African populations. Methods: In this cross-sectional study, placental parenchyma specimens with limited membrane sampling were collected from 103 Ivorian and Ghanaian mothers without known pregnancy or birth complications. Tissue pieces adjacent to PCR-tested samples were analyzed by real-time PCR targeting *Chlamydia trachomatis*, *Mycoplasma hominis*, *Neisseria gonorrhoeae*, *Schistosoma* spp., *Streptococcus agalactiae*, *Trichomonas vaginalis*, *Ureaplasma parvum* and *Ureaplasma urealyticum.* Corresponding adjacent tissues were examined by routine histopathology, supplemented with immunohistochemistry when higher pathogen DNA quantities were detected, to assess inflammatory changes. Results: Real-time PCR detected *U. urealyticum* in 15 out of 103 cases (14.6%, ±0.7%), *U. parvum* in 13 (12.6%, ±0.6%), *S. agalactiae* in 11 (10.7%, ±0.5%), the *S. haematobium* complex in four (3.9%, ±0.2%), *M. hominis* in four (3.9%, ±0.2%), confirmed *N. gonorrhoeae* in two (1.9%, ±0.1%) and non-confirmed *N. gonorrhoeae* in one (1.0%, ±0.1%), *T. vaginalis* in two (1.9%, ±0.1%), and *C. trachomatis* (non-lymphogranuloma venereum serovar) in one (1.0%, ±0.1%). Overall, pathogen DNA levels were low, with only four positive PCR results yielding cycle threshold (Ct) values below 30 and none below 25. Histopathological examination revealed no relevant inflammatory changes in any samples. Conclusions: Placental parenchyma tissues with limited membrane sampling testing positive for *Schistosoma* spp. or sexually transmitted pathogens by molecular methods demonstrated no corresponding histopathological inflammation. These findings warrant confirmatory studies to better characterize potential region-specific placental infection phenotypes and their clinical significance.

## 1. Introduction

Placental infections constitute a significant health and survival risk for fetuses in West Africa [1,2]. Several sexually and non-sexually transmitted microorganisms have been associated with adverse pregnancy outcomes.

Among regionally prevalent non-sexually transmitted infectious agents, *Schistosoma* spp. warrant particular attention in West Africa. Increased rates of low birth weight, prematurity, and stillbirth have been observed in cases of maternal schistosomiasis, and proinflammatory responses in maternal, placental, and fetal compartments have been linked to these outcomes [3,4,5]. In particular, soluble egg antigens of *Schistosoma* spp. have been shown to activate proinflammatory molecular pathways in placental trophoblasts [6]. However, the sensitivity of histological examinations for diagnosing genital schistosomiasis in placental tissue is limited by the low density and uneven distribution of helminth eggs [3,7]. Notably, even adult worms have occasionally been detected histologically within the intervillous space and decidual vessels adjacent to eggs in severe cases [5]. Although tissue maceration techniques have been proposed to improve diagnostic sensitivity, they remain laborious and unsuitable for routine diagnostics [3]. Importantly, no evidence of fetal infection has been reported even in severe cases of placental schistosomiasis [5].

Associations between sexually transmitted microorganisms and pregnancy complications are well established, with substantial data available for *Mycoplasma* spp. and *Ureaplasma* spp. More than four decades ago, transcervical migration of these organisms from the lower genital tract to the placenta was described and associated with polymorphonuclear infiltration of the placental membranes, fetal surface, and umbilical cord [8,9]. Detection of *Mycoplasma* and *Ureaplasma* species in placental tissue, whether indicative of colonization or infection, has been associated with preterm delivery, chorioamnionitis, fetal infection, and stillbirth [10,11,12]. In particular, *Ureaplasma parvum* has been implicated in placental infection [13], while a study in a low-risk Latvian population reported increased risk of intrauterine infection with *U. parvum* only in the presence of co-infections [14]. In neonates, detection of *Mycoplasma* spp. has been associated with increased risk of bronchopulmonary dysplasia [10].

With regard to the localization of placental inflammation, *Mycoplasma* spp.-associated infection tends to involve the membranes [10], although findings on chorionic plate involvement of *Mycoplasma* spp. and *Ureaplasma urealyticum* remain conflicting [10,15]. *Ureaplasma* spp. colonization has been linked to infertility, stillbirth, and histologic chorioamnionitis in mothers, as well as congenital pneumonia, bronchopulmonary dysplasia, meningitis, and perinatal death in neonates [16,17,18,19,20,21,22]. In preterm infants, colonization of the respiratory tract with *Ureaplasma* spp. has been associated with necrotizing enterocolitis [23]. Recently, *U. urealyticum* and *Gardnerella vaginalis* were detected in amniotic fluid and associated with intra-amniotic inflammation in patients with clinical chorioamnionitis at term [24], contrasting with earlier reports suggesting that chorioamnionitis at term is primarily non-infectious [25]. In smaller studies from Mexico and China, *U. urealyticum* was the most frequently detected microorganism associated with intrauterine infection [26,27]. Experimental evidence suggests that the severity of *U. parvum*-associated intrauterine infection may depend on host genetic predisposition [28]. Although both organisms are considered to have low virulence, the strength of their associations with pregnancy complications remains debated [10]. Quantitative rather than qualitative differences in microbial abundance—also observed in healthy uterine and chorionic tissues —have been associated with preterm delivery and gestational age [29,30]. *Mycoplasma* and *Ureaplasma* species are detected more frequently in placental samples from the second trimester rather than the first, whereas such differences are not observed for viral agents such as herpes simplex virus and cytomegalovirus [31]. In late pregnancy, *U. urealyticum* and *U. parvum* show low transmission rates to the placenta and fetus, although *U. parvum* has been linked to postpartum endometritis [32].

*Streptococcus agalactiae* has been associated with an increased risk of spontaneous mid-gestation abortion [33,34]. Experimental studies have demonstrated that this bacterium can induce chorioamnionitis with marked polymorphonuclear infiltration [35] and that *S. agalactiae*-induced choriodeciduitis can progress to intra-amniotic infection associated with preterm labor [36]. In contrast, histologic evidence of fetoplacental inflammation has been a poor predictor of perinatal *S. agalactiae* infection [37].

Historical investigations have suggested no association between *Chlamydia trachomatis* and stillbirth or abortion [38,39], and significant colonization of amniotic membranes with *C. trachomatis* has not been observed [40]. Similarly, *Neisseria* species are rarely detected in placental inflammatory lesions [41]. *Trichomonas vaginalis* has been associated with vaginal dysbiosis during pregnancy [42] and may facilitate ascending bacterial infections leading to complications [43]. Although these three microorganisms have occasionally been isolated from the amniotic cavity following preterm delivery, their etiological relevance remains uncertain [44].

Beyond sexually transmitted pathogens, vaginal dysbiosis-associated *G. vaginalis*, anerobic bacteria such as *Bacteroides* spp., and enteropharyngeal organisms including members of the order Enterobacterales, as well as *Haemophilus influenzae* and *Staphylococcus aureus*, have been linked to pregnancy complications and preterm labor [45]. Nevertheless, estimates of intra-amniotic infection based on placental culture and histopathology remain uncertain [46]: the accuracy of placental culture in predicting amniotic fluid infection ranges from 44% to 57%, and placental histopathology identifies intra-amniotic inflammation in only 58% of cases [46]. Notably, male preterm infants are more likely to exhibit positive placental membrane cultures and increased decidual lymphoplasmacytic infiltration, suggesting more pronounced maternal immune activation [47]. Overall, the association between infectious chorioamnionitis and preterm birth appears moderate in magnitude [48]. Regarding fetal death, maternal inflammation seems to play a more determinative role than fetal inflammatory responses [49].

This study aims to contribute to existing knowledge on the associations between pathogen detection and placental inflammatory alterations by assessing spatial correlations between molecular pathogen detection and histopathological evidence of placental inflammation for selected sexually transmitted microorganisms and *Schistosoma* spp. Adjacent placental tissue samples were analyzed using real-time PCR and traditional histopathological staining. The samples were obtained from a cohort of West African mothers without known pregnancy or delivery complications.

## 2. Materials and Methods

### 2.1. Study Type and Population as Well as Inclusion and Exclusion Criteria

This study was conducted as a comparative analysis using fully anonymized human sample material. Placental tissue samples were collected immediately after delivery from Ivorian and Ghanaian mothers participating in the Child Development Study (CDS), which investigates the effects of communicable and non-communicable diseases on infant development in sub-Saharan Africa, as described in detail elsewhere [50,51]. In Côte d’Ivoire, samples were collected at Abobo Community Hospital in Abidjan, and in Ghana at Komfo Anokye Teaching Hospital in Kumasi. The inclusion criteria for this analysis were a placental tissue weight exceeding 200 mg and the absence of amplification inhibition in real-time PCR. Exclusion criteria comprised maternal risk factors or pregnancy complications. Specifically, volunteers were excluded in cases of maternal age below 18 years, multiple pregnancy, diabetes, hypertension, hemorrhage, preeclampsia, preterm delivery, stillbirth, or low birth weight.

### 2.2. Sampling

Placental samples were collected immediately after delivery from randomly selected areas of the tissue. Specimens were fixed in 100% ethanol and stored at −80 °C. At the assessment sites in Germany, samples were divided into two parts: approximately 200 mg of tissue was used for molecular analysis, and the directly adjacent portion was processed for histopathological examination.

### 2.3. Molecular Diagnostics and Associated Case Definitions

The EZ1&2 DNA tissue kit protocol (Qiagen, Hilden, Germany) was applied on EZ1 automatic nucleic acid extractors (Qiagen, Hilden, Germany) according to the manufacturer’s instructions for DNA extraction from the respective 200 mg tissue volumes. Before starting the protocol, bead-beating-based tissue lysis was performed using 3.5 mm steel beads in liquid-nitrogen frozen tubes for 5 min at 30/s using a TissueLyser LT device (Qiagen, Hilden, Germany). After nucleic acid extraction, DNA (desoxyribonucleic acid) in the eluates was quantified using a Pico 100 Picodrop microliter spectrophotometer (Picodrop Ltd., Hinxton, UK) according to the manufacturer’s instructions, yielding a mean ± standard deviation (SD) of 237.7 ng/µL ± 84.4 ng/µL. Eluates were deep-frozen at −80 °C prior to PCR analyses.

Real-time PCR protocols obtained from the literature [52,53,54,55,56,57,58,59,60,61] were applied to screen for DNA of *C. trachomatis*, *M. hominis*, *Neisseria gonorrhoeae*, *S. agalactiae*, *Schistosoma* spp., *T. vaginalis*, *U. parvum*, and *U. urealyticum*. Details on target sequences, diagnostic accuracy estimates from the literature [52,53,54,55,56,57,58,59,60,61], and technical detection limits as determined with 10-fold dilution series of positive control plasmids containing the assay target sequences are provided in Table 1.

The assays were run on RotorGene Q cyclers (Qiagen, Hilden, Germany), each run contained a plasmid-based positive control and a PCR grade water-based negative control. Typical sigmoid-shaped amplification curves were accepted as most likely target-specific, and no cut-off was applied with regard to measured cycle threshold (Ct) values. Semi-quantification based on Ct values was performed using the categories “high pathogen density” for Ct values < 20, “intermediate pathogen density” for Ct values ≥ 20 but <30, and “low pathogen density” for Ct values ≥ 30.

Because low pathogen densities close to the technical detection limits of the PCRs were considered as likely, at least two different real-time PCRs per target microorganism were applied. For diagnostic case definitions, each typically shaped real-time PCR signal was considered a true positive. In line with previous recommendations [53], both *N. gonorrhoeae* PCRs had to be positive to define a confirmed case, whereas a non-confirmed case was defined as a situation with only one out of two real-time PCR assays testing positive. The two applied PCRs for *C. trachomatis* targeted a *C. trachomatis* cryptic plasmid sequence for species-specific screening and the *pmpH* gene for discrimination of serovars A–K from serovars L1–L3 [54]. More specifically, the *pmpH*-based serovar discrimination used a pan-serovar-specific hybridization probe and a serovar A–K-specific probe. Accordingly, an L1–L3 serovar was diagnosed if the pan-serovar-specific hybridization probe, but not the A–K-specific probe, showed a positive fluorescence signal [54]. Finally, the two *Schistosoma* spp.-specific PCRs allowed discrimination of the *S. mansoni* complex, indicated by a positive result of the *Sm1-7* sequence-specific PCR, and the *S. haematobium* complex, indicated by a positive *Dra1*-specific PCR [52]. A phocid herpesvirus DNA-specific real-time PCR was performed with each sample to exclude sample inhibition [62]. In line with the standards of the diagnostic laboratory, a plasmid containing the phocid herpesvirus target sequence [62] was spiked into each sample prior to nucleic acid extraction at a quantity resulting in a Ct-value range between 25 and 30. To control the risk of sample contamination during laboratory procedures, strict adherence to three-room-separation of nucleic acid extraction, master mix preparation, and nucleic acid amplification was ensured. In addition, sample series were monitored for suspicious patterns of neighboring samples showing positive real-time PCR results with increasing Ct values as potential indicators of contamination transferred between adjacent specimens.

### 2.4. Histopathological Workup and Comparison with the Molecular Diagnostic Results

Randomized tissue samples were thawed at room temperature in a bath of 100% ethanol and transferred to capsules. Fixation was performed in 6% formaldehyde for at least 5 days. Subsequent processing was completed within one week, resulting in formaldehyde exposure for five-twelve days. All tissue specimens were paraffin-embedded, sectioned at approximately 5 µm thickness, and stained with hematoxylin and eosin (H&E; Morphisto, Offenbach am Main, Germany).

Histopathological evaluation focused on inflammatory changes and, where applicable, followed the Amsterdam Placental Workshop Group Consensus Statement criteria [63]. Assessment was performed independently and in a blinded fashion by two pathologists experienced in placental pathology. After the initial evaluation and subsequent unblinding, a histopathological re-evaluation was conducted. Additionally, samples with a real-time PCR Ct value below 30 were recut and subjected to immunohistochemical staining to detect macrophages and neutrophilic granulocytes using anti-CD15 (clone Carb3) and anti-CD68 (clone KP1) antibodies (Agilent, Santa Clara, CA, USA). Representative images were captured using an Axio Imager. Z2 microscope (Zeiss, Jena, Germany) equipped with an Axiocam 305 color camera (Zeiss, Jena, Germany).

### 2.5. Statistics

Given the moderate sample size and low frequency of pathogen detections, study results were presented descriptively as part of this hypothesis-generating, exploratory analysis. Because no prior assumptions were made regarding expected findings or effect sizes, no sample size calculation was performed; instead, all available specimens were included in the analysis.

### 2.6. Ethics

All study procedures complied with the Declaration of Helsinki and its subsequent amendments. Ethical approval for the Child Development Study (CDS) was obtained from the National Ethics Committee of Côte d’Ivoire (Ref: 4169/MHSP), the Ethics Committee of the Kwame Nkrumah University of Science and Technology in Kumasi, Ghana (Ref: CHRPE/KNUST/KATH/01_06_08), and the Ethics Committee of the Hamburg Chamber of Physicians, Germany (Ref: PV3020). Written informed consent for participation was obtained from all CDS participants.

## 3. Results

### 3.1. Characterization of the Study Population

As summarized in Table 2, the study population consisted predominantly of Ivorian mothers, with only a small proportion from Ghana. The mean maternal age was slightly below 30 years, and on average, placental samples were obtained from the third delivery. Most births occurred via the vaginal route. Consistent with the exclusion of complications during pregnancy and delivery, the mean APGAR score at one minute exceeded 8 and was close to 9 at five minutes. The majority of participating mothers had limited formal education, were employed in occupations not requiring specialized training, and lived under modest socioeconomic conditions. Household amenities such as flush toilets or freezers were uncommon and considered luxuries rather than standard equipment.

### 3.2. Molecular Proof of Microorganisms in the Placental Samples

As detailed in Appendix A Table A1, the molecular diagnostic assays demonstrated 15/103 (14.6%, ±0.7%) detections of *U. urealyticum*, 13 (12.6%, ±0.6%) detections of *U. parvum*, 11 (10.7%, ±0.5%) detections of *S. agalactiae*, four (3.9%, ±0.2%) detections of *S. haematobium* complex, four (3.9%, ±0.2%) detections of *M. hominis*, two (1.9%, ±0.1%) confirmed detections and one (1.0%, ±0.1%) non-confirmed detection of *N. gonorrhoeae*, two (1.9%, ±0.1%) detections of *T. vaginalis*, and one (1.0%, ±0.7%) detection of a *C. trachomatis* serovar not associated with lymphogranuloma venereum, in declining order of frequency (Table 3). In nine of 103 (8.7%, ±0.4%) placental samples, more than one microorganism was detected, including five cases of co- detection of *U. parvum* and *U. urealyticum*, one case of *U. parvum* and *M. hominis*, one case of *S. agalactiae* with non-confirmed *N. gonorrhea*, one case with *U. parvum*, *M. hominis* and *U. urealyticum*, and one case with *U. parvum*, *U. urealyticum* and *S. agalactiae*. As also shown in Appendix A, Table A1, and summarized in Table 3, low quantities of microbial DNA with cycle threshold values ≥ 30 were observed, with four exceptions: two detections of the *cfb* gene of *S. agalactiae*, one detection of the 67-base pair region within a multicopy sequence in the *T. vaginalis* genome, and one detection of the *Dra1* sequence of *S. haematobium* complex. Even in these four instances, Ct values were >25. In a total of 11 cases, both applied target-specific PCRs were positive, thereby confirming the diagnosis. These comprised six infections with *S. agalactiae*, two with *N. gonorrhoeae*, and one each with *C. trachomatis* non-L1-3, *M. hominis*, and *U. parvum* (Table 3). Of note, the two *S. agalactiae* infections with Ct values < 30 for the *cfb* gene were among these 11 confirmed cases.

### 3.3. Histopathological Assessment

**Figure 1:**
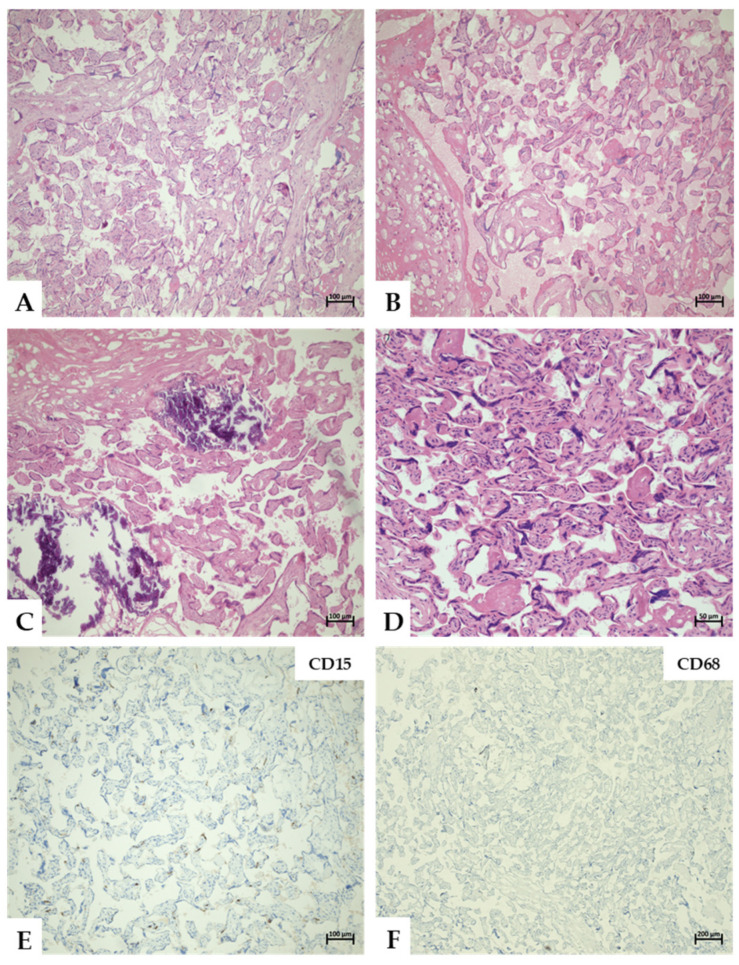
Representative histological findings within the assessed placental tissues. (**A**–**D**) Representative images of mature placental parenchyma of the third trimester, showing numerous terminal villi, some intermediate villi, and a few stem villi. Minor fibrin depositions were observed in the basal decidua (**B**) along with sparse calcifications (**C**), both considered normal signs of minor regression. Hematoxylin and eosin (H&E) staining. (**E**,**F**) Representative immunohistochemical staining of sample 19: CD15-specific antibodies highlight loosely distributed immature endothelial cells and nonspecific autolytic cell debris without evidence of neutrophil granulocytes (**E**). CD68-specific antibodies show no accumulation of macrophages or histiocytes (**F**). Magnifications are 50× in (**F**), 100× in (**A**–**C**,**E**), and 200× in (**D**). Scale bars represent 50 µm, 100 µm, and 200 µm.

All 103 tissue samples were suitable for histological evaluation after refixation in formaldehyde. The majority of samples consisted predominantly of placental parenchyma with portions of decidua (*n* = 62/103; 60.2%, ±3.0%); in many samples, only placental parenchyma was present (*n* = 33/103; 32.0%, ±1.6%), whereas amnion was detectable in only a minority of samples (*n* = 8/103; 7.8%, ±0.4%). While 60–100% of DNA detections per assessed microbial species were associated with the presence of decidua tissue, only a single detection of *S. agalactiae* DNA occurred in a sample containing amnion tissue (Table 3). No tissue sample contained both decidua and amnion. In all cases, placental maturity corresponded to the third trimester. No signs of inflammation, helminth tissue, or helminth eggs were identified in any specimen. After re-evaluation and immunohistochemical staining, no additional pathology was detected. Because inflammatory changes were entirely absent, staging and grading according to the Amsterdam scheme were not performed. Representative findings are shown in Figure 1.

**Table 3:** Comparison of molecular pathogen detection and histology proof of membranes in adjacent tissue.

Detected Microorganism	Proportion of Positive Samples (*n*/*n*, Percentage, 95% Confidence Interval)	Number of Positive Results Confirmed by Another Assay	Number of Positive Samples Indicating “Intermediate Pathogen Density” Defined as Ct Value ≥ 20 but <30 with at Least One Assay	Number of Positive Samples Indicating “Low Pathogen Density” Defined as Ct Value ≥ 30 with Any Target-Specific Assay	Number of PCR-Positive Samples Showing Amnion in Histology	Number of PCR-Positive Samples Showing Decidua in Histology
*Chlamydia trachomatis*	1/103 (1.0%, ±0.7%)	1	0	1	0	1
*Mycoplasma hominis*	4/103 (3.9%, ±0.2%)	1	0	4	0	3
*Neisseria gonorrhoeae*	3/103 (1.9%, ±0.1%)	2	0	3	0	2
*Ureaplasma parvum*	13/103 (12.6%, ±0.6%)	1	0	13	0	9
*Ureaplasma urealyticum*	15/103 (14.6%, ±0.7%)	0	0	15	0	9
*Schistosoma haematobium*	4/103 (3.9%, ±0.2%)	n.a.	1	3	0	3
*Streptococcus agalactiae*	11/103 (10.7%, ±0.5%)	6	2	9	1	8
*Trichomonas vaginalis*	2/103 (1.9%, ±0.1%)	0	1	1	0	2

*n* = number. n.a. = not applicable.

## 4. Discussion

This study aimed to assess histopathological signs of inflammation in placental tissue adjacent to samples used for DNA detection of *Schistosoma* spp. and sexually transmitted microbial agents. Notably, no relevant inflammatory changes were observed, which holds several implications.

Regarding placental schistosomiasis, low helminth egg density is a recognized challenge that limits the diagnostic sensitivity of conventional histopathology [3,7]. To address this, the study analyzed tissue adjacent to PCR-positive samples, hypothesizing that soluble egg antigens released from deposited *Schistosoma* eggs might induce detectable inflammation [6]. Two hypotheses may explain the absence of inflammation: first, PCR positivity might reflect single, sparsely distributed eggs causing minimal local inflammation undetectable even in nearby sections. This is plausible given the high sensitivity of the *Dra1* assay [52]. Second, PCR might detect circulating cell-free *Schistosoma* DNA within placental blood vessels, consistent with the assay’s diagnostic design. Both interpretations affirm the high sensitivity of *Dra1*-based real-time PCR in detecting schistosomiasis with few or absent tissue eggs, supported by elevated Ct values.

The absence of inflammation despite molecular detection of *Mycoplasma* spp. and *Ureaplasma* spp.—traditionally linked to placental inflammation [8,9]—is consistent with all detections showing high Ct values (>30), indicating to low pathogen density. Earlier studies report that inflammation when caused by these species depends on pathogen load and that these organisms can also be harmless colonizers [29]. These findings align with prior reports from uncomplicated pregnancies in West African populations [29].

Similarly, *S. agalactiae* and *T. vaginalis*, detections at intermediate pathogen densities (Ct 20–30) showed no histological inflammation. Low detection rates of *N. gonorrhoeae* and *C. trachomatis* reflect their rarity in placental tissue [40,41]. Given the overall low microbial DNA loads, the possibility of secondary contamination during vaginal delivery cannot be excluded, especially since only one confirmed cesarean section was part of the study and pathogen DNA was not detected in that sample (data not shown).

Notably, Ct value-based semi-quantification as performed in our study requires careful interpretation; therefore, only approximations over Ct value ranges were provided. As reported elsewhere [64], measured inhibition control Ct values obtained with fixed placental tissues showed a standard deviation range corresponding to approximately two orders of magnitude of target DNA concentration. This partial sample inhibition, combined with probable effects of sample storage and transfer from the African study sites, prevented attempts at absolute quantification. Furthermore, although some PCR targets occurred in multiple copies per pathogen genome, as described for *Schistosoma* spp.-specific assays [52], the actual sensitivity gain achievable with these assays is substantially less than expected due to the multicopy nature of the target sequences [65]. As elaborated elsewhere [65], unpredictable sequence variations within those repeat regions are responsible for this phenomenon. Focusing on target sequence quantity per sample, rough estimations of sequence numbers based on diagnostic threshold values assessed with dilution series of positive control plasmids (data not shown) suggest that “high pathogen density”, defined as Ct values < 20, corresponds to target DNA copy numbers exceeding 10 million; “intermediate pathogen density”, defined as Ct values ≥ 20 but <30, corresponds to a range between 10 thousand and 10 million; and “low pathogen density”, defined as Ct values ≥ 30, corresponds to fewer than 10 thousand copies.

This study has several limitations. First, by design, there was no control group of mothers with pregnancy or delivery complications [50,51]. Consequently, while the current analysis demonstrates microbial DNA detection without histopathologic inflammation in uncomplicated pregnancies, conclusions regarding pathological pregnancies cannot be drawn. Second, the PCR protocols employed were not optimized specifically for placental tissue, raising uncertainty about whether the diagnostic accuracy reported for other sample types applies here. Particularly given the low measured Ct values and the low inter-assay concordance of positive real-time PCR results, confirmatory testing strategies beyond use of alternative assays for the same parameters would have been advisable. Because such additional approaches were not implemented, only 11 positive PCR results could be confirmed by two independent methods, whereas the remainder must be considered unconfirmed by an independent assay. Third, distinction among microbial colonization, true infection, and contamination during vaginal delivery was not feasible. Contamination with DNA of sexually transmitted microorganisms during vaginal delivery likely accounts for the absence of inflammation in at least some cases, especially given the low amounts of detected target DNA. Fourth, placental specimens were collected randomly without systematic sampling of defined regions; as a result, amnion tissue was often absent, precluding the histopathological identification of chorioamnionitis. Fifth, limited tissue availability restricted the number of comparative assessments in this exploratory analysis, therefore minor effects may have gone undetected. Sixth, the sample origin was unevenly distributed between the Ghanaian and Ivorian sites, preventing conclusions on regional epidemiology. Finally, available epidemiological information on participants lacked data on maternal antibiotic exposure near delivery and maternal comorbidities that could suppress inflammation. These factors might have influenced the findings, and the absence of such data should be regarded as a potential source of bias.

## 5. Conclusions

Despite these limitations, this exploratory hypothesis-generating study demonstrated absent or minimal inflammatory reactions in placental parenchyma tissue, with only limited membrane sampling, from uncomplicated pregnancies adjacent to samples testing positive for *Schistosoma* spp. and sexually transmitted microorganisms by molecular methods at low target DNA quantities. Future confirmatory assessments are necessary to substantiate or refute specific regional phenotypes. Such studies should include robust sample size calculations, adequate control groups, systematic membrane sampling, and application of multi-level, quality-controlled molecular diagnostic approaches to comprehensively characterize placental infection and inflammatory profiles in endemic settings.

## Figures and Tables

**Table 1:** Characteristics of the applied microbial target-specific real-time PCRs assays. Diagnostic accuracy estimates were derived from published evaluation studies. Technical detection limits were determined using 10-fold serial dilution of positive control plasmids containing the target sequences.

Microbial Target	Target Sequence of Real-Time PCR 1; Sensitivity in %, Specificity in %, Technical Detection Limit in Copies/µL	Target Sequence of Real-Time PCR 2; Sensitivity in %, Specificity in %, Technical Detection Limit in Copies/µL	References
*Chlamydia trachomatis*	*pmpH*; copy-number depending sensitivity close to 100% in combination with PCR 2 ^c^, 100% specificity in combination with PCR 2 ^c^, <10^2^ copies/µL	*C. trachomatis* cryptic plasmid sequence; copy-number depending sensitivity close to 100% in combination with PCR 1 ^c^, 100% specificity in combination with PCR 1 ^c^, <10^2^ copies/µL	[54]
*Mycoplasma hominis*	*tuf* gene; 97.0% sensitivity ^f^, 99.5% specificity ^e^, <10^1^ copies/µL	*yidC* gene; 100% sensitivity ^g^, 100% specificity ^g^, <10^2^ copies/µL	[53]
*Neisseria gonorrhoeae*	*opa* gene; 100% sensitivity (in combined use with *porA* gene assessment) ^b^, 99.3% specificity (in combined use with *porA* gene assessment) ^b^, <10^2^ copies/µL	*porA* gene; 100% sensitivity (in combined use with *opa* gene assessment) ^b^, 99.3% specificity (in combined use with *opa* gene assessment) ^b^, <10^2^ copies/µL	[56,57]
*Schistosoma* spp.	*Sm1-7* (multicopy target occurring in about 60,000 copies per *S. mansoni* complex genome); 93.3% sensitivity ^a^, 100% specificity ^a^, <10^3^ copies/µL	*Dra1* (multicopy target occurring in about 20,000 copies per *S. haematobium* complex genome); 95.9% sensitivity ^a^, 97.3% specificity ^a^, <10^3^ copies/µL	[52]
*Streptococcus agalactiae*	*cfb* gene; 100% sensitivity ^h^, 100% specificity ^e^, <10^2^ copies/µL	*sip* gene; 97.0% sensitivity ^i^, 99.0% specificity ^i^, <10^2^ copies/µL	[58,59]
*Trichomonas vaginalis*	67-base pair region within a multi-copy sequence in the *T. vaginalis* genome; 100% sensitivity ^d^, 99.6% specificity ^d^, <10^1^ copies/µL	sequence of the beta-tubulin protein; 100% sensitivity ^d^, 99.9% specificity ^d^, <10^1^ copies/µL	[60,61]
*Ureaplasma parvum*	*ureD* gene; 100% sensitivity ^d^, 100% specificity ^e^, <10^1^ copies/µL	*clpB* gene; 97.0% sensitivity ^f^, 99.5% specificity ^e^, <10^1^ copies/µL	[55,56]
*Ureaplasma urealyticum*	ABC transporter permease gene; 95.6% senstitivity ^f^, 96.9% specificity ^e^, <10^1^ copies/µL	*ureD* gene; 100% sensitivity ^d^, 100% specificity ^e^, <10^1^ copies/µL	[55,56]

^a^ in serum; ^b^ in urogenital and pharyngeal swabs; ^c^ in rectal swabs; ^d^ in genital swabs; ^e^ tested with DNA of non-target microorganisms; ^f^ in urine, genital swabs, fluid and tissue samples; ^g^ in genital, perianal and anal swabs; ^h^ tested with target organisms and pharyngeal swabs; ^i^ in vaginal and rectal swabs. µL = microliter.

**Table 2:** Characterization of the study population (*n* = 103).

Age and Country of Origin of the Pregnant Women	
Mean age ± standard deviation (SD)	28.4 (±5.8)
Country of origin: number (percentage)	Ivory Coast: *n* = 100 (97.1%, ±4.9%) Ghana: *n* = 3 (2.9%, ±0.1%)
Pregnancy- and birth-related information	
Mean number of pregnancies ± standard deviation (SD) ^1^	3.4 (±2.0)
Type of delivery: number (percentage)	Vaginal delivery: *n* = 100 (97.1%, ±4.9%) Section: *n* = 1 (1.0%, ±0.1%) Missing datasets: *n* = 2 (1.9%, ±0.1%)
Mean APGAR 1 value ± standard deviation (SD) ^2^	8.1 (±0.8)
Mean APGAR 2 value ± standard deviation (SD) ^2^	8.8 (±0.6)
Socio-economic characterization	
Education level: number (percentage)	None: *n* = 42 (40.8%, ±2.0%) Basic: *n* = 37 (35.9%, ±1.8%) Secondary: *n* = 20 (19.4%, ±1.0%) Tertiary: *n* = 4 (3.9%, ±0.2%)
Kind of occupation: number (percentage)	Housewife: *n* = 27 (26.2%, ±1.3%) Farmer: *n* = 1 (1.0%, ±0.1%) Trader: *n* = 26 (25.2%, ±1.3%) Salery worker: *n* = 9 (8.7%, ±0.4%) Other: *n* = 40 (38.8%, ±1.9%)
Source of water: number (percentage)	Piped water: *n* = 103 (100%, ±5.0%)
Kind of toilet: number (percentage)	Pit latrine: *n* = 70 (68.0%, ±3.4%) Improved pit latrine: *n* = 25 (24.3%, ±1.2%) Flush toilet: *n* = 8 (7.8%, ±0.4%)
Kind of floor in the household: number (percentage)	Earth/sand: *n* = 4 (3.9%, ±0.2%) Vinyl/tiles: *n* = 12 (11.7%, ±0.6%) Cement: *n* = 87 (84.5%, ±4.2%)
Electricity in the household: number (percentage)	No: *n* = 3 (2.9%, ±0.1%) Yes: *n* = 100 (97.1%, ±4.9%)
Freezer in the household: number (percentage)	No: *n* = 78 (75.7%, ±3.8%) Yes: *n* = 25 (24.3%, ±1.2%)
Farming of poultry close to the household: number (percentage)	No: *n* = 85 (81.6%, ±4.1%) Yes: *n* = 18 (17.5%, ±0.9%)

^1^ 9 datasets missing. ^2^ 1 dataset missing.

## Data Availability

All relevant data are provided either in the manuscript or Appendix A.

## Abbreviations

The following abbreviations are used in this manuscript:

*C.t.*	*Chlamydia trachomatis*
*C.t.* non-L1-L3	*Chlamydia trachomatis* without the serovars L1-L3
CDS	Child Development Study
Ct	cycle threshold
DNA	desoxyribonucleic acid
ID	anonymized sample number
*M.h.*	*Mycoplasma hominis*
µL	Microliter
n	Number
n.a.	Not applicable
*N.g.*	*Neisseria gonorrhoeae*
PCR	polymerase chain reaction
SD	standard deviation
*S.a.*	*Streptococcus agalactiae*
*S.h.*c.	*Schistosoma haematobium* complex
*S.m.*c.	*Schistosoma mansoni* complex
spp.	species (plural)
T.v.	*Trichomonas vaginalis*
*U.p.*	*Ureaplasma parvum*
*U.u.*	*Ureaplasma urealyticum*

## Appendix A

**Table A1:** Distribution of the recorded real-time PCR signals over the assessed placenta samples. The headers of the table indicate the target microorganisms and the target sequences are in the row below. Negative PCR signals are indicated in white and positive ones in gray, including the measured cycle threshold (Ct) values shown as plain numbers. The samples are fully anonymized and denoted with plain numbers only.

ID	*S.m.*c.	*S.h.*c.	*C.t.* Non-L1-L3	*C.t.*	*N.g.*	*N.g.*	*T.v.*	*T.v.*	*U.p.*	*U.p.*	*S.a.*	*S.a.*	*M.h.*	*M.h.*	*U.u.*	*U.u.*
	*Sm1-7*	*Dra1*	*pmpH*	*Chlamydia trachomatis cryptic plasmid sequence*	*Opa*	*porA*	67-base pair region within a multi-copy sequence in the *T. vaginalis* genome	sequence of the beta-tubulin protein	*ureD*	*clpB*	*cfb*	*sip*	*tuf*	*yidC*	ABC transporter permease gene	*ureD*
1																
2																
3																
4																
5																
6									34						35	
7																
8															35	
9										37						
10																
11																
12																
13																
14																
15																
16												38				
17											31	34				
18								35								
19							29									
20																
21									34	36			34	37	37	
22										35						
23																
24											31	34				
25										33						
26																
27																
28					32							39				
29																
30			32	30												
31																
32										35					36	
33																
34																
35												38				
36																
37																
38															37	
39																
40											33	37				
41																
42																
43																
44																
45																
46																
47																
48											33	39				
49															37	
50																
51												39				
52																
53										32				34		
54																
55																
56											28	31				
57																
58															37	
59									31			37			32	
60																
61																
62																
63																
64																
65																
66																
67															36	
68																
69																
70																
71										38						
72					33	32										
73																
74														38		
75																
76																
77																
78																
79															37	
80															37	
81																
82									33						33	
83																
84																
85																
86																
87																
88											26	30				
89																
90																
91									34						36	
92															37	
93														37		
94										35					38	
95																
96									35							
97																
98	29															
99	32															
100	32															
101	34															
102																
103					33	31										

ID = anonymized sample number; *S.m.*c. = *Schistosoma mansoni* complex; *S.h.*c. = *Schistosoma haematobium* complex; *C.t.* non-L1-L3 = *Chlamydia trachomatis* without the serovars L1-L3; *C.t.* = *Chlamydia trachomatis*; *N.g.* = *Neisseria gonorrhoeae*; *T.v.* = *Trichomonas vaginalis*; *U.p.* = *Ureaplasma parvum*; *S.a.* = *Streptococcus agalactiae*; *M.h.* = *Mycoplasma hominis*; *U.u.* = *Ureaplasma urealyticum*.
